# Internal Ring Defect Closure Technique in Laparoscopic Mesh Hernioplasty for Indirect Inguinal Hernia

**DOI:** 10.3389/fsurg.2022.794420

**Published:** 2022-02-07

**Authors:** Binggen Li, Shange Shi, Changfu Qin, Jiwei Yu, Duhui Gong, Xiangyang Nie, Jinchao Miao, Zeru Lai, Wenbo Cui, Guoxin Li

**Affiliations:** ^1^Department of General Surgery, Affiliated Hexian Memorial Hospital of Southern Medical University, Guangzhou, China; ^2^Department of General Surgery and Guangdong Provincial Key Laboratory of Precision Medicine for Gastrointestinal Tumor, Nanfang Hospital, The First School of Clinical Medicine, Southern Medical University, Guangzhou, China; ^3^Department of General Surgery, Pengpai Memorial Hospital, Shanwei, China; ^4^Department of Hernia and Abdominal Wall Surgery, Beijing Chaoyang Hospital, Capital Medical University, Beijing, China; ^5^Department of General Surgery, Shanghai Ninth People's Hospital, School of Medicine, Shanghai Jiao Tong University, Shanghai, China

**Keywords:** indirect hernia, internal ring defect closure, laparoscopic hernioplasty, scrotal hernia, seroma, TAPP

## Abstract

**Purpose:**

The best way to reduce seroma formation after laparoscopic indirect hernia repair is debated. We noticed that internal ring defect closure in laparoscopic mesh hernioplasty could provide promising outcomes with an effect on diminishing seroma formation. We introduce our closure technique and report our experience.

**Methods:**

This prospective study was conducted from May 2019 to May 2021. Patients with European Hernia Society classification L3 indirect or scrotal hernia were recruited and underwent laparoscopic transabdominal patch plasty (TAPP). Hernia defect closure was performed before mesh deployment. The primary outcomes were seroma formation, postoperative pain, and hernia recurrence. Perioperative data and postoperative complications were also recorded.

**Results:**

Consecutive 77 patients with 89 indirect hernias (including 51 scrotal hernias) were recruited in two regional tertiary hospitals. All operations were successful without open conversion. The mean size of the hernia defect was 3.7 ± 0.5 cm (range, 2.5–5.0 cm). The mean operative time for each hernia repair (peritoneum to peritoneum) was 48.3 ± 10.8 min (range, 33–72 min), and the mean time required for internal ring closure was 6.7 ± 2.2 min (range, 4–10 min). Intraoperative bleeding was minimal. The mean visual analog scale pain score at rest on the first postoperative day was 2.2 (range, 1–4). The average postoperative length of hospital stay was 18 h (range, 14–46 h). During a mean follow-up period of 9.4 months (range, 3–23 months), no hernia recurrence or chronic pain were noted. Seroma formation was detected on six sides of unilateral hernias (6.7%) on postoperative day 7, with a mean volume of 45.8 ml (range, 24–80 ml). All seromas were mild and resolved spontaneously within 3 months, with no need for evacuation or other treatment and without major impact on the final outcome.

**Conclusions:**

Defect closure in laparoscopic mesh hernioplasty for large indirect hernias is safe and feasible and can significantly reduce postoperative seroma formation and relative complications. This approach is recommended in large indirect or scrotal hernia repair.

Laparoscopic inguinal hernia repair (LIHR), including endoscopic total extraperitoneal patch plasty (TEP) and laparoscopic transabdominal patch plasty (TAPP) techniques, has become one of the gold standards for inguinal hernia repair. These techniques have been adopted globally owing to the merits of minimal invasiveness and high effectiveness. A large-scale, registry-based, comparative study showed that TEP and TAPP have advantages over the Lichtenstein procedure (1). However, these techniques require a longer learning curve than for open surgery, and some postoperative complications are worrisome. Seroma formation is common after hernia operation, particularly in medial defects, large indirect hernias, or scrotal hernias (2–4). The incidence of seroma in scrotal hernia varies from 10.5 to 70% (3, 4). Although most seromas are considered a frequent minor postoperative morbidity, with no impact on recovery (4), some refractory cases develop an infection that leads to eventual repair failure (5).

For direct hernias, there is clear evidence to support the finding that the seroma incidence can decrease significantly when the lax transversalis fascia is inverted (6), which means that the incidence of seroma formation could be reduced by eliminating dead space. For large indirect hernias (European Hernia Society classification (EHS) L3) or scrotal hernias, methods of reducing seroma formation remain undefined. Some researchers found that patients undergoing indirect hernia sac transection were more likely to develop postoperative seroma compared with those undergoing complete sac dissection (7, 8), suggesting dissecting the indirect hernia sac as completely as possible to reduce seroma formation (8). However, other researchers revealed that hernia sac transection is simple and effective, reduces the operative time, and does not increase the rate of seroma formation (9). Fan et al. reported that early postoperative (23 h) preperitoneal drainage can effectively decrease seroma formation and proposed this drainage for patients who are susceptible to postoperative seroma formation (10). Daes suggested pulling and fixing the distal sac to the lateral posterior inguinal wall in large inguinoscrotal hernias, believing that this technique could reduce the chance of seroma formation (11). On the other hand, Liu et al. revealed that, compared with blunt dissection, monopolar energy device dissection could further decrease the incidence of seroma formation (12). Overall, tremendous effort has been spent to resolve this issue, but none has come to an exact and effective end.

We have also made various attempts to investigate this dilemma. In addition to the above-mentioned measures, we noticed that internal ring defect closure in laparoscopic mesh hernioplasty could provide promising outcomes, with its effect on diminishing seroma formation. We introduce this technique and report our positive experience with its application, in this study.

## Materials and Methods

This prospective study was conducted in the Affiliated Hexian Memorial Hospital of Southern Medical University and Pengpai Memorial Hospital from May 2019 to May 2021. The study was approved by the institutional ethical review board of each hospital. The main surgeons who participated in this study were experienced experts who had each performed more than 300 TAPP procedures. The inclusion criteria were indirect inguinal hernia classified as L3 according to the EHS classification (defect size ≥3 cm, including primary and recurrent cases) (13). Most scrotal hernias are large indirect hernias (L3) that represent a major surgical challenge and are associated with a high probability of postoperative seroma; therefore, this scenario was included in our study. All patients were in healthy preoperative general condition without significant comorbidities and were able to tolerate pneumoperitoneum and general anesthesia. The exclusion criteria were patients who complained of preoperative groin pain in the scrotal area, combined hernias (e.g., pantaloon hernia), strangulated hernia, loss of domain, and absolute clinical contraindications (e.g., active intra-abdominal infection or abdominal wall fistula).

Preoperatively, all patients underwent dynamic ultrasound Doppler examination to verify the hernia defect and obtain preliminary measurements of the hernia sac volume. A complete medical history was obtained, and a thorough physical examination was performed to identify any abnormalities that could affect the surgery. All patients provided informed consent preoperatively. The patients' demographics, hernia defect size, sac volume, intraoperative findings, operative details (the internal ring closure time consuming was recorded exclusively), and perioperative and postoperative data were collected prospectively. The main outcomes of the study were seroma formation, postoperative pain, hernia recurrence, and intraoperative/postoperative complications. Complications, namely hematoma, infection, wound dehiscence, or any serious adverse events, were recorded and analyzed. Continuous variables are presented as mean ± standard deviation, and categorical variables are presented as number and percentage.

## Seroma Formation Definition and Measurement

1) Clinically palpable, irreducible, swelling without cough impulse in the groin and scrotal area.
2) Fluid collection in the distal sac (groin and scrotal area) detected by ultrasonography.

The seroma size was measured by Doppler ultrasonography, and sac volume was calculated using the formula for an ellipsoid [V = (4/3) Π abc], where a, b, and c represent the radius of the length, width, and height, respectively).

## Operative Technique

The operation was performed under general anesthesia. Considering the operative time was relative short, then no antibiotics were used, and no urinary catheter was placed preoperatively. The main procedures were similar to those in standard guidelines (14), and in the following description, we omitted the common techniques that surgeons are familiar with. However, the main subject of internal ring closure and adjunctive techniques are described in detail.

In this series, we mainly transected the hernia sac instead of complete dissection (only one patient was undergone hernia sac complete dissection in our series. The hernia sac of this patient was wide and shallow, and was very easy to dissect completely). After the primary dissection of the indirect hernia sac, we transected the sac from the top to the base. Care should be taken to preserve the underlying cord structures during dissection. The dissection process was mainly performed using mono-polar devices (e.g., electric hook, scissors, or forceps); no harmonic scalpel or other energy devices were required.

**Figure 1:**
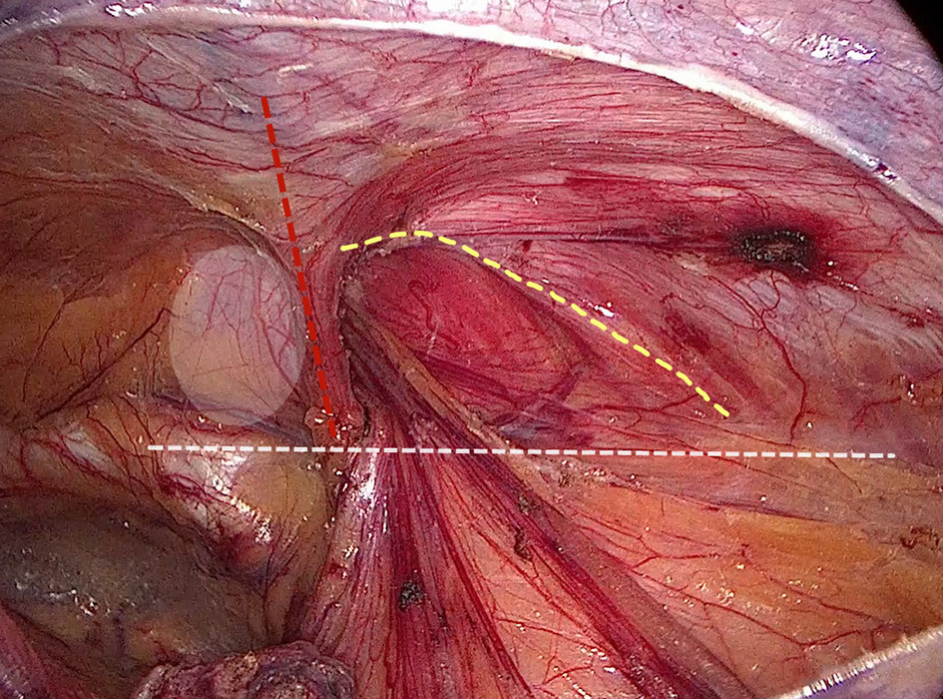
Surgical field after space dissection and sac management. (Red dashed line: epigastric vessels; hatched area: transversalis fascia; white dashed line: ilio-pubic tract; yellow dashed line: conjoined tendon).

**Figure 2:**
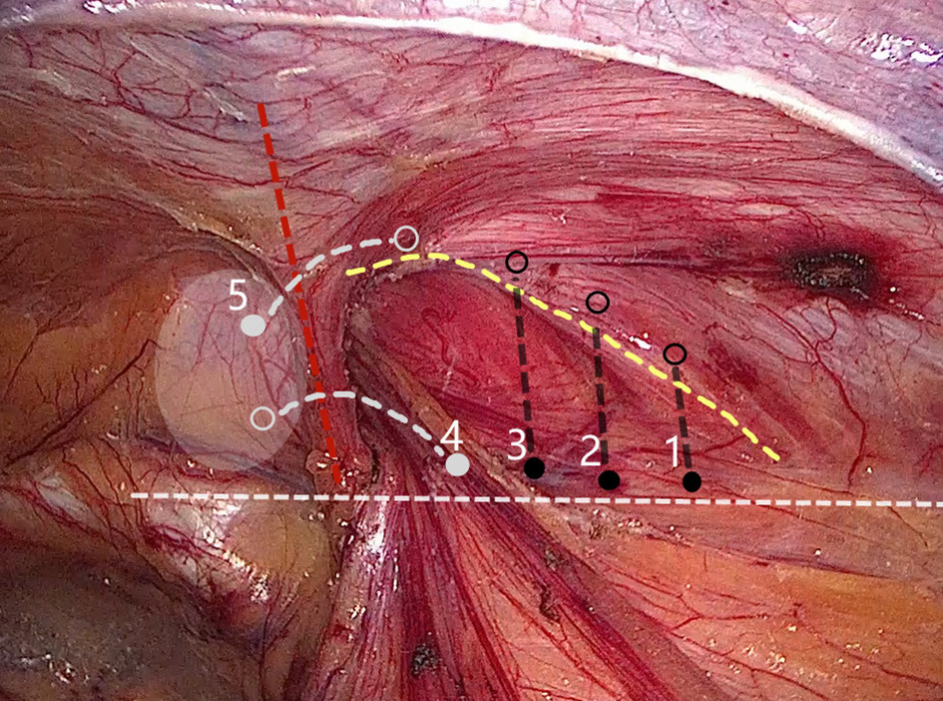
Internal ring closure using a five-stitches method. (Solid dot: needle entry; open dot: needle extraction).

**Figure 3:**
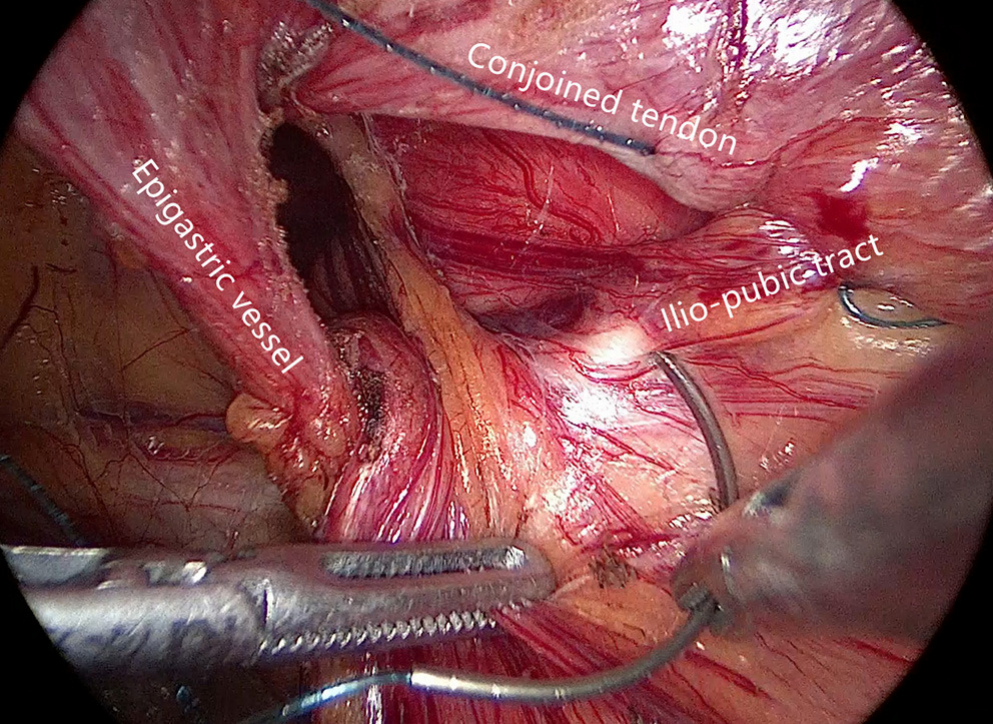
The ilio-pubic tract and conjoined tendon were sutured in a running fashion.

**Figure 4:**
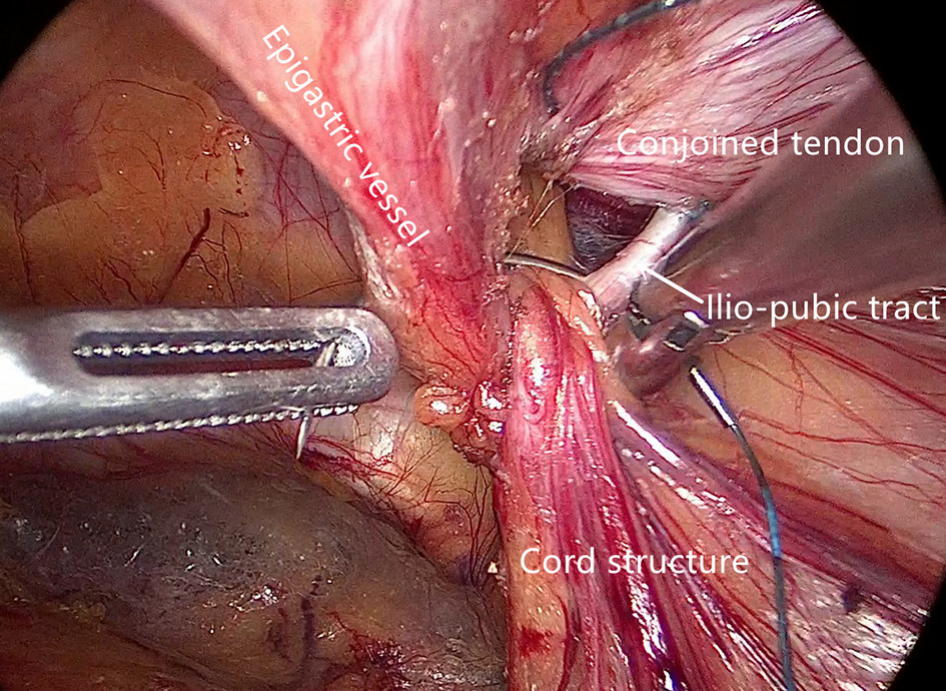
The fourth stitch.

**Figure 5:**
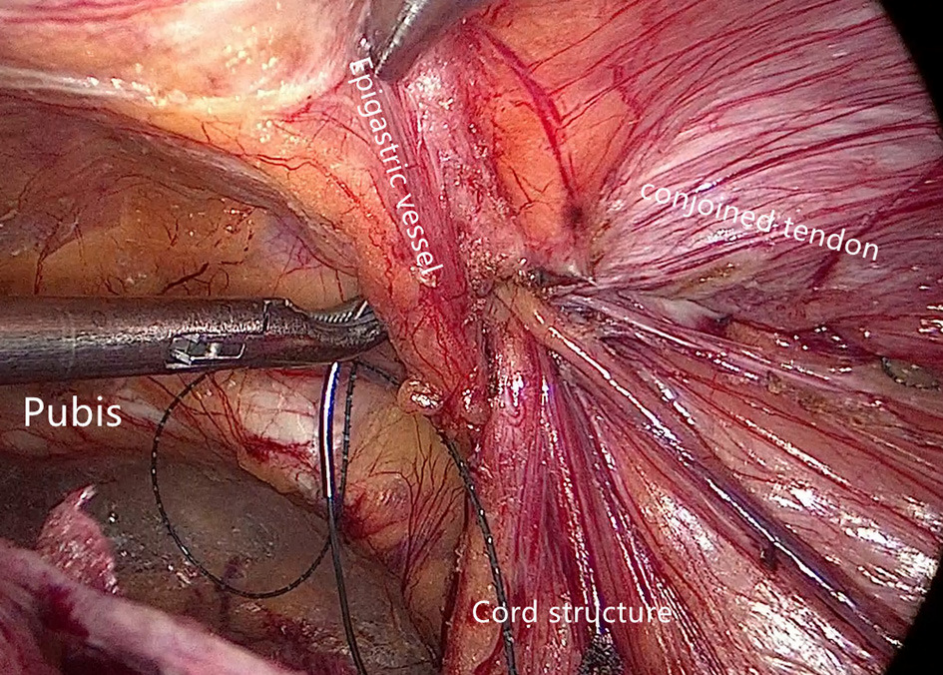
The fifth stitch.

**Figure 6:**
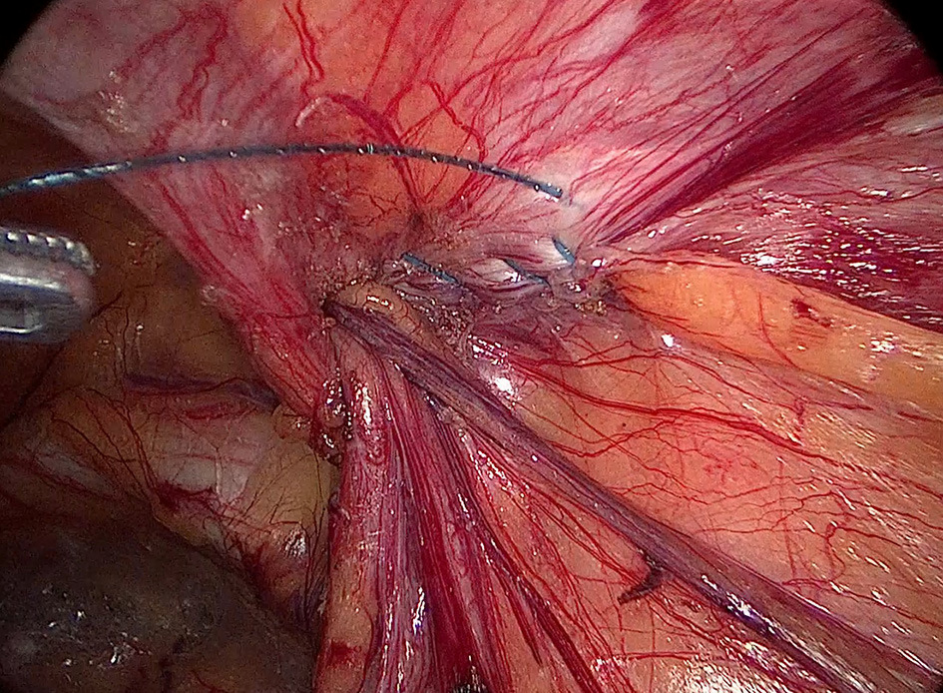
The closed internal ring.

Internal ring closure. After the space dissection and sac management (Figure 1) and before mesh placement, we closed the internal ring using a five-stitches method (Figure 2). A 2-0 barbed, slowly-absorbable, suture (VLOCL0315, Covidien llc, Mansfield, MA, USA) was used. Suturing proceeded from lateral to medial by backward suturing manner, the ilio-pubic tract and the conjoined tendon were sutured in a running fashion. The needle advancing direction was perpendicular to the ilio-pubic tract, and the suture bites were small and superficial to minimize the chance of underlying vessel (iliac artery) and nerve [femoral branch of the genitofemoral nerve (GFN)] injury. The suture bite on the conjoined tendon also should be shallow to avoid potentially jeopardizing the iliohypogastric nerve (IHN) and ilioinguinal nerve (IIN), which run in the inguinal box. The intersuture spacing was ~8–10 mm. Generally, after three continuous stitches (Figure 3), suturing approached the epigastric vessels, which is when we placed the last two sutures in a U-shape. Fourth continuous stitch: After taking a small bite in the ilio-pubic tract, we advanced the needle above the cord, then penetrated the transversalis fascia behind the epigastric vessels, into the medial aspect of the vessel (Figure 4). Fifth continuous stitch: At a distance of 1 cm from the previous needle extraction point, the needle penetrated the transversalis fascia behind the vessels again, then we took a small bite of the conjoined tendon, and the needle was extracted and returned to the lateral aspect of the epigastric vessels (Figure 5). After tightening the suture, the ilio-pubic tract, conjoined tendon, and transversalis fascia behind the vessels were pulled together, achieving the goal of internal ring closure (Figure 6).

Some details of this technique should be noted. Before beginning to suture, surgeons should carefully examine the possible femoral branch of the GFN, which may run on the surface of the iliac muscle, and take precautions, if this branch is present. An appropriate path should be left for the cord structures, without compressing or leaving a large gap. A simple way to determine whether the tightness of the closure is appropriate is, after closure, if the cord is still retractable, there is no compression. Additionally, after squeezing the gas out of the distal sac, if there is no refilling, the closure is sufficient. Finally, if one U-shaped stitch is insufficient, surgeons can place additional sutures until the path for the cord structures is appropriate.

**Figure 7:**
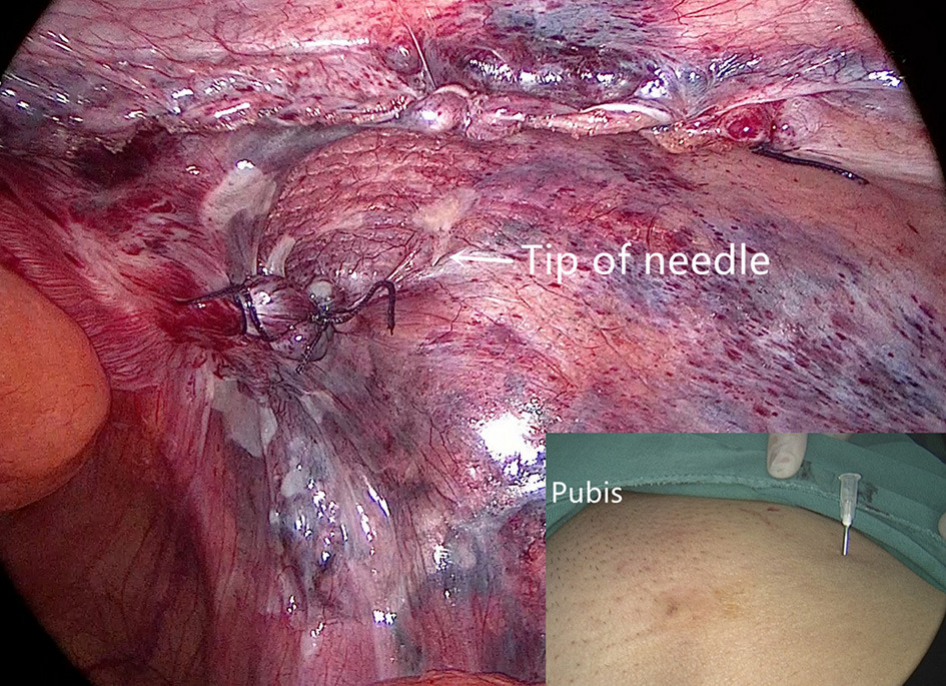
The peritoneum and mesh are pressed against the abdominal wall after thick needle deflation.

We used a 3D macroporous partially absorbable polypropylene mesh (15 cm × 10 cm) (PASL; TransEasy Medical Tech Co., Ltd., Beijing, China) for the repair. No drainage and no fixation (glue or tacks) were performed in our series. To ensure the mesh was pressed against the abdominal wall after deflation, we used the additional technique of thick needle deflation. Under pneumoperitoneum, after completing the peritoneal closure, we inserted a thick needle (1.6-mm caliber) into the preperitoneal space, which was already accommodating the mesh. Under the pressure of pneumoperitoneum, the peritoneum and the 3D mesh were pressed against the abdominal wall (Figure 7). Finally, we removed the trocars and discontinued pneumoperitoneum under direct vision.

## Results

This 2-year prospective study involved 77 patients (61 men and 16 women; age range, 25–87 years) and 89 indirect hernias. Among them, 81 were primary hernias, and 8 were recurrent; there were 51 scrotal hernias. All operations were successfully performed by the first and second authors without serious intraoperative complications. No open conversion was required. Table 1 shows the demographic and perioperative data. The mean size of the hernia defect was 3.7 ± 0.5 cm (range, 2.5–5.0 cm). The mean operative time for each hernia repair (either side; peritoneum to peritoneum) was 48.3 ± 10.8 min (range, 33–72 min), and the mean time for internal ring closure was 6.7 ± 2.2 min (range, 4–10 min). Intraoperative bleeding was minimal. Postoperative pain was measured and recorded on postoperative day 1 and was qualitatively mild with a mean visual analog scale pain score at rest of 2.2 ± 0.8 (range, 1–4). We encouraged early ambulation and resumption of an oral diet under the concept of enhanced recovery after surgery (ERAS). This TAPP approach was mainly completed as day surgery; therefore, the average postoperative length of hospital stay was 18 h (range, 14–46 h).

**Table 1:** Patients' general demographics and perioperative data.

**Variable**	**TAPP for indirect hernia**
N (male/female)	77 (61/16)
Age (years)	61.2 ± 13.7
BMI (kg/m2)	22.3 ± 3.1
Hernia sides sum (primary/recurrent/scrotal)	89 (81/8/51)
Hernia defect size (cm)	3.7 ± 0.5
Operative time (mins)^*^	48.3 ± 10.8
Time for internal ring closure (mins)	6.7 ± 2.2
VAS pain score^*^	2.2 ± 0.8
LOS (hours)	18 ± 3.7
Seroma formation (volume)	6 sides (45.8 ± 19.9 ml)
Follow-up (months)	9.4 ± 5.7

*BMI, body mass index; VAS, visual analog scale; LOS, length of postoperative stay*.

* *Operative time was recorded for each side, from peritoneum to peritoneum*.

* *VAS pain score was obtained at rest on postoperative day 1*.

All patients were instructed to return for a clinical examination 1 week after the surgery. Routine physical examination was performed to detect any early postoperative complications (e.g., postoperative ileus, infection, urinary retention, severe pain, wound occurrence, hematoma, or seroma). If patients complained of an irreducible swelling/mass in the groin and scrotal area, ultrasonography was performed to further differentiate seroma, hematoma, or distal sac edema. Fluid collection in the distal sac without a Doppler signal and > 10 ml in volume was interpreted as seroma-positive. If patients reported no complaints, they were followed-up by a telephone interview every 2 months. If a problem was identified during the telephone interview, the patient was requested to visit the clinic for further examination.

By September 2021, 72 patients (93.5%) had been followed-up for a mean period of 9.4 ± 5.7 months (range, 3–23 months). No hernias recurred, and no severe early postoperative pain or chronic refractory pain was noticed during the follow-up. No patients developed postoperative ileus, wound-related complications, mesh infection, or postoperative hematoma. Regarding seroma formation, six sides of unilateral indirect hernias developed seromas (6.7%), with a mean volume of 45.8 ± 19.9 ml (range, 24–80 ml). These patients reported no complaints or only a mild feeling of scrotal swelling. All seromas resolved spontaneously within 3 months and required no puncture and evacuation.

## Discussion

Internal ring closure under laparoscopy is not novel; this approach is simply an old wine in a new bottle. In our study now, we try to endow this technique with some new meanings. As early in 1990, Ger et al. (15) had initiated animal studies of laparoscopic internal ring closure to treat indirect hernia. Later, in the 1990s, researchers performed this technique in humans (16–18). Indirect hernias were repaired using only pure suture closure of the internal ring instead of mesh-based repair. The preliminary results were promising, with a reported recurrence rate of 0–3%, and complications were rare (16–18). However, most surgeons consider this closure technique suitable only for small indirect hernias in young patients who are unlikely to have a weak inguinal floor. Furthermore, compared with the overall recurrence rate of TAPP/TEP repair at <1% (17), the results of pure suture closure remain imperfect. Therefore, pure laparoscopic internal ring closure for inguinal hernia repair has not been widely accepted or implemented.

**Figure 8:**
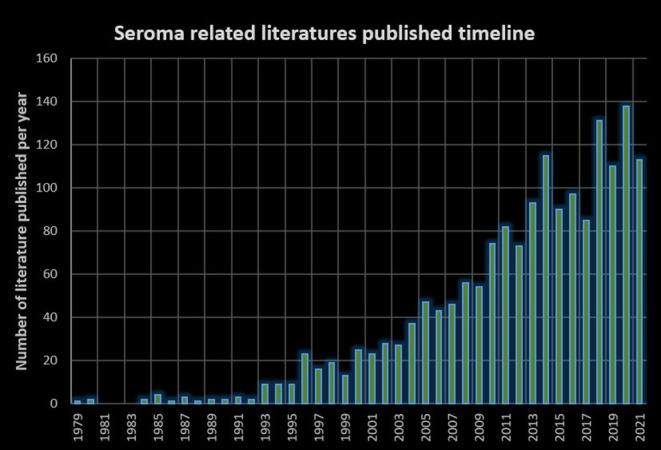
Seroma-related published literature timeline.

Seroma is a relatively new topic. Looking back to the open surgery era, seromas were rarely mentioned, and the word “seroma” rarely appeared in studies involving the Lichtenstein technique (19, 20). As a matter of fact, postoperative seroma is a new issue that emerged after the popularization of laparoscopic hernia repair. PubMed searches of the timeline of seroma-related publications confirm this trend (Figure 8).

Despite the fact that most seromas resolve spontaneously and have no impact on the final convalescence, seromas significantly and negatively impact patients' medical experiences, leading to misconceptions or even medical lawsuits. Reducing the postoperative seroma rate is a substantial subject that deserves further intensive research. For voluminous direct hernias, substantial evidence has shown that the incidence of seromas can be significantly reduced when the extended transversalis fascia is inverted (6). However, for large indirect or scrotal hernias, the definitive method of seroma reduction is undetermined. Some researchers proposed complete dissection of the indirect hernia sac; however, other researchers reported the contrary conclusion that hernia sac dissection increases seroma risk and provides no benefit. Preperitoneal drainage was once considered a promising measure to reduce seromas. Fan et al. reported that early short-term preperitoneal drainage effectively decreased the overall incidence of seroma formation (10). We postulate that early temporary drainage could break the vicious circle of exudation-aseptic inflammation-exudation. However, in our previous practice, drainage did not affect seroma incidence. During the early stage postoperatively (<7 days) with a drainage tube *in situ*, we did not, indeed, observe seromas; however, once the drain was removed, seromas emerged. We hypothesize that this phenomenon is attributable to the short duration of drain placement (shorter than the wound exudate stage); therefore, a prolonged drainage period (2–3 weeks) may deserve further research. Some researchers suggest regional compression or ice compresses, which require that patients lie flat in the early postoperative stage. This recommendation is based on individual experience and not high-level evidence. Moreover, these methods compromise patients' early ambulation and potentially increase the risk of thromboembolism (21).

Currently, there is still no confirmed method of seroma prevention after indirect hernia repair. In fact, we believe that seroma formation is inevitable, as wound exudation certainly occurs. Internal ring closure does not eliminate seromas (exudation fluid) but simply restricts it in the preperitoneal space where it is non-palpable and imperceptible, instead of being located in the distal sac, where it is obvious and uncomfortable. The preperitoneal space is endowed with rapid healing and absorptive properties; therefore, seromas (exudation fluid) could be absorbed over a certain period (we anticipate 1–2 weeks). However, seromas in the distal sac remain for a long period, as we have encountered. We expect that the distal sac has a poor blood supply and very limited exudate absorption ability. Some prime examples could indirectly support our standpoint. Open inguinal hernia repairs are rarely associated with seromas, and we postulate that internal ring closure contributes to this result. In large indirect hernias, regardless of Lichtenstein repair or open preperitoneal repair, the widened internal ring is tightened or closed (20, 22). Another example is pediatric indirect hernia high ligation, in which the distal sac is never manipulated, however, one could hardly detect a postoperative seroma or hydrocele. We postulate from these facts that seromas originate mainly from upstream preperitoneal space exudation instead of distal sac secretions.

Another merit of internal ring closure is transforming indirect hernia surgery into a reinforcement repair. Formerly, surgeons would leave the defect patent and perform repair only with mesh coverage, which is a bridging repair. For small to medium defects (<3 cm), this weakness could be counteracted by sufficient overlapping of the mesh. However, in accordance with Laplace's law (23), as defects increase in size, the chance of mesh protrusion increases, and this weakness is enhanced by heavy-weight mesh or fixation measures. We don't deny that internal ring closure will bring the repair with tension. However, relatively speaking, inguinal defect is small, the closure of this defect will not produce much tension. Besides, internal ring closure could turn a bridging repair into a reinforcement repair, which makes the repair more reliable and theoretically lowers the recurrence rate. Moreover, other repair materials could be taken into options, such as light-weight mesh or biological mesh.

**Figure 9:**
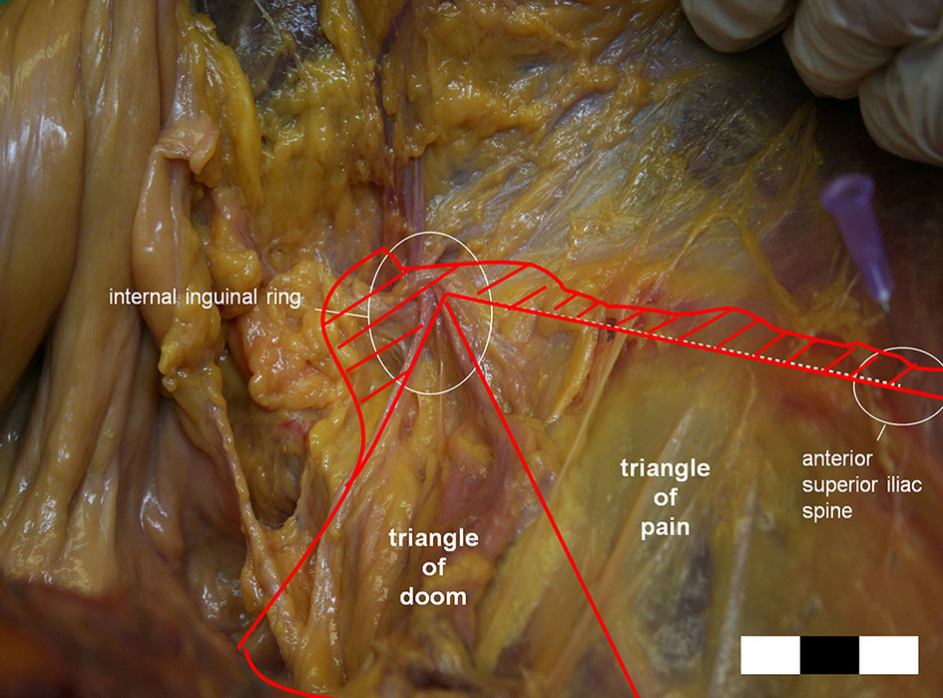
The possible area of the genitofemoral nerve (GFN) or lateral femoral cutaneous nerve (LFCN) injury is larger than previously reported. (Hatched area: extension of the triangles of doom, and pain.) (Courtesy of Prof. Wolfgang Reinpold).

We believe the major concern with our internal ring closure technique is the risk of nerve and vessel injuries. There are some vital structures around the internal ring, namely the cord structures, epigastric vessels, iliac artery, and branches of the GFN, and suturing in this location is extremely dangerous; however, we believe these dangers are avoidable. Vessels and cord structures are constant and apparent and easy to avoid, but nerves are not easily visible and vary in location. According to Reinpold et al. (24), the anatomical area of possible nerve injury [GFN and lateral femoral cutaneous nerve (LFCN)] is even larger than previously reported, as shown in Figure 9. On the other hand, the IHN and IIN travel in the inguinal box, which is superficial to the internal ring (25). Considering these nerves' variability and the spatial dimensions of the nerves' paths can prevent direct nerve injury and subsequent inguinodynia (26). In our practice of internal ring closure, we implemented the five-stitches method, which was always adhere to nerve protect strategy. Moreover, we used an absorbable suture material, which minimized long-term irritation. We believe these are the reasons why there was no postoperative severe early or chronic pain in our cohort.

The results of our study are promising. Compared with our previous practice, the postoperative seroma incidence decreased significantly. The previous bridging repair was transformed into a reinforcement repair, making the surgery more reliable, and more types of mesh could be selected (e.g., a lightweight mesh could be used in this scenario to decrease the foreign body material remaining in the body). Additionally, patients required no restriction of ambulation, rapidly returned to their daily activities, and experienced a lower rate of thromboembolism-related complications. Despite the slightly increased operative time, no obvious intraoperative or postoperative complications were observed. In particular, the most concerning complications of nerve injury and pain did not occur, which was quite encouraging. In general. The comfort scale of these patients was significantly improved.

In conclusion, our internal ring closure technique in large indirect hernia TAPP repair is safe and feasible. Mastering the suture skills and knowledge of nerve anatomy could minimize related complications. However, seroma formation is a multifactorial issue that cannot be completely resolved by only one measure. Additionally, our study is a preliminary exploration with no control group and a short follow-up period. Therefore, the strength of our study is limited. The true efficacy of this technique should be explored in larger scale, multicenter, randomized controlled trials.

## Data Availability Statement

The original contributions presented in the study are included in the article/supplementary material, further inquiries can be directed to the corresponding author.

## Ethics Statement

The studies involving human participants were reviewed and approved by the Ethical Review Board of Affiliated Hexian Memorial Hospital and the Ethical Review Board of Pengpai Memorial Hospital. The patients/participants provided their written informed consent to participate in this study.

## Author Contributions

All authors listed have made a substantial, direct, and intellectual contribution to the work and approved it for publication.

## Funding

This work was supported by the Guangdong Provincial Key Laboratory of Precision Medicine for Gastrointestinal Cancer (2020B121201004), the Guangdong Provincial Major Talents Project (No. 2019JC05Y361), and WU JIEPING Medical Foundation Clinical Research Project (No. 320.6750.18393).

## Conflict of Interest

The authors declare that the research was conducted in the absence of any commercial or financial relationships that could be construed as a potential conflict of interest.

## Publisher's Note

All claims expressed in this article are solely those of the authors and do not necessarily represent those of their affiliated organizations, or those of the publisher, the editors and the reviewers. Any product that may be evaluated in this article, or claim that may be made by its manufacturer, is not guaranteed or endorsed by the publisher.
